# Optimizing illumina next-generation sequencing library preparation for extremely at-biased genomes

**DOI:** 10.1186/1471-2164-13-1

**Published:** 2012-01-03

**Authors:** Samuel O Oyola, Thomas D Otto, Yong Gu, Gareth Maslen, Magnus Manske, Susana Campino, Daniel J Turner, Bronwyn MacInnis, Dominic P Kwiatkowski, Harold P Swerdlow, Michael A Quail

**Affiliations:** 1 Wellcome Trust Sanger Institute, Hinxton, Cambridge, CB10 1SA, UK; 2 Oxford Nanopore Technologies, Edmund Cartwright House, 4 Robert Robinson Avenue, Oxford OX4 4GA, UK

**Keywords:** Next-Generation Sequencing, Illumina, Library, *Plasmodium falciparum*, AT-rich, Malaria, Clinical isolate, PCR, Tetramethyammonium chloride, PCR-free, Isothermal, Linear, Exponential

## Abstract

**Background:**

Massively parallel sequencing technology is revolutionizing approaches to genomic and genetic research. Since its advent, the scale and efficiency of Next-Generation Sequencing (NGS) has rapidly improved. In spite of this success, sequencing genomes or genomic regions with extremely biased base composition is still a great challenge to the currently available NGS platforms. The genomes of some important pathogenic organisms like *Plasmodium falciparum *(high AT content) and *Mycobacterium tuberculosis *(high GC content) display extremes of base composition. The standard library preparation procedures that employ PCR amplification have been shown to cause uneven read coverage particularly across AT and GC rich regions, leading to problems in genome assembly and variation analyses. Alternative library-preparation approaches that omit PCR amplification require large quantities of starting material and hence are not suitable for small amounts of DNA/RNA such as those from clinical isolates. We have developed and optimized library-preparation procedures suitable for low quantity starting material and tolerant to extremely high AT content sequences.

**Results:**

We have used our optimized conditions in parallel with standard methods to prepare Illumina sequencing libraries from a non-clinical and a clinical isolate (containing ~53% host contamination). By analyzing and comparing the quality of sequence data generated, we show that our optimized conditions that involve a PCR additive (TMAC), produces amplified libraries with improved coverage of extremely AT-rich regions and reduced bias toward GC neutral templates.

**Conclusion:**

We have developed a robust and optimized Next-Generation Sequencing library amplification method suitable for extremely AT-rich genomes. The new amplification conditions significantly reduce bias and retain the complexity of either extremes of base composition. This development will greatly benefit sequencing clinical samples that often require amplification due to low mass of DNA starting material.

## Background

Among the eukaryotic pathogens whose genomes have so far been sequenced, the malaria parasite, *Plasmodium falciparum *(*P. falciparum*), posed the most technical challenges both in sequencing and assembly of the draft genome [1]. The biggest hurdle was the complexity of the genome, which is extremely base biased. The *P. falciparum *genome is very AT-rich; coding regions have a mean AT content of more than 75%, with up to 100% in intergenic and non-coding regions [2]. Although biased base composition has been a challenge to the Sanger sequencing methods, it has continued to be a major problem for the current high-throughput Next-Generation Sequencing (NGS) technologies [3]. Much work has focused on solving amplification and sequencing problems associated with high GC content but nothing has been done to improve on those caused by AT-rich parts of the genome [4,5]. Since completion of the malaria genome sequencing and the subsequent development of massively parallel sequencing technology, there has been increased effort in using genetic information obtained through re-sequencing initiatives to control and ultimately eliminate malaria (Manske *et al*, submitted). The Wellcome Trust Sanger Institute is currently using Illumina NGS technologies to re-sequence thousands of clinical isolates from various malaria endemic regions, mainly sourced from Africa and Asia. Re-sequencing of these samples has faced two major challenges: low mass of total parasite DNA and extremely high AT-base composition of the genome. These problems pose major technical challenges predominantly in the library preparation stage of the NGS pipeline and also in subsequent data analysis. Due to extreme AT bias, standard Illumina library preparation procedures produce libraries with poor representation that are generally low in complexity compared to the genomic DNA from which they were derived. Polymerase chain reaction (PCR) amplification conditions as currently used in the standard library preparation procedures, have been shown to introduce biases in sequence coverage towards DNA regions with balanced base composition[5,6]. Furthermore, genomic regions with high AT or very high GC content generally result in little or no amplification at all. These PCR-introduced artifacts have been shown to cause misleading or inaccurate conclusions in the analysis of genome-variation data [4].

Attempts have been made to overcome coverage problems associated with PCR amplification by omitting the PCR step in library preparation[6]. Although the PCR-free approach has improved read distribution and produced more even genome coverage, it requires large amounts of starting DNA material, which is difficult to obtain especially from clinical isolates. The low mass of DNA in clinical isolates demands some form of amplification to generate sufficient quantities for an Illumina library preparation. We have therefore investigated various amplification possibilities and developed library-preparation procedures that are optimal for extremely AT-rich genomes using the Illumina sequencing platform.

Here we report alternative library-amplification procedures optimized for AT-rich templates and suitable for low mass DNA starting material especially from malaria clinical isolates. We have tested various library amplification methods including linear and isothermal amplification strategies. We have also explored various PCR amplification conditions by testing different polymerases, and their tolerance to AT-rich templates, in the absence or presence of tetramethylammonium chloride (TMAC), a PCR additive that increases thermostability of AT base pairs [7].

## Results

### Conventional PCR amplification of an AT-rich locus

**Figure 1:**
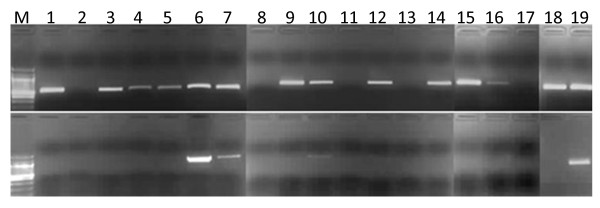
**Screening for tolerance to an AT-rich template using conventional PCR amplification**. Top panel: PCR amplification of a 540 bp locus (Pf3D7_11:1294982-1295521) with a relatively balanced (70% AT) base composition (positive control) in the presence or absence of TMAC. Bottom panel: PCR amplification of a 1217 bp locus (Pf3D7_01:55900-57116) with extreme AT content (84%) in the presence or absence of TMAC. M, 100 bp DNA ladder (NEB); (1) PWO master; (2) PWO master + TMAC; (3) PfuULTRA; (4) PfuULTRA + TMAC; (5) Kapa HiFi; (6) Kapa HiFi + TMAC; (7) AccuPrime Taq HiFi; (8) AccuPrime Taq HiFi + TMAC; (9) AccuPrime pfx SuperMix; (10) Phusion; (11) Phusion +TMAC; (12) Platinum HiFi; (13) Platinum HiFi + TMAC; (14) Platinum pfx; (15) Platinum pfx + TMAC, (16) Ex Taq; (17) Ex Taq + TMAC; (18) Kapa2G Robust; (19) Kapa2G Robust + TMAC.

In order to identify polymerases and PCR conditions that are efficient and tolerant to high AT content templates, we performed conventional PCR using commercial enzymes. The PCR was targeted at a 1217 bp locus (Pf3D7_01:55900-57116) with a high AT-content (84%) and a 540 bp locus (Pf3D7_11:1294982-1295521) with a relatively balanced base composition (positive control) from the *P. falciparum *3D7 genome http://www.plasmodb.org[8]. Pf_1 and Pf_11_ama1 oligo pairs (see additional file 1, Table S1) were used to amplify the high AT-rich and positive control fragments respectively. For each enzyme, two sets of PCRs were performed, one using reagents and conditions provided by the manufacturer (standard conditions) and the other deviating only by inclusion of 60 mM TMAC in the reaction mixture (optimized conditions). TMAC is a DNA-binding reagent that has been shown to increase the melting temperature of AT base pairs, thereby improving thermostability of the AT-rich regions during PCR amplification. Although in the presence of 3M TMAC, the thermostability of AT base pairs is equal to GC base pairs, this concentration is also inhibitory to polymerase activity. It has been shown that low concentrations of TMAC (60 mM) improve AT base pair stability and result in increased specificity and yield of PCR products [7]. As shown in Figure 1, most enzymes tested were able to amplify the relatively balanced base composition fragment in the absence of TMAC additive, although with different efficiencies. The presence of TMAC inhibited the activity of most enzymes except for Kapa HiFi, Kapa2G Robust and Platinum pfx, where the additive had a positive effect in amplifying the base-balanced region. At the extreme AT-rich region, nearly all of the enzymes tested were unable to produce detectable amplification either in the presence or absence of TMAC additive. AccuPrime Taq HiFi and Phusion enzymes produced low but detectable amplification of the test region only in the absence of TMAC. However, Kapa HiFi and Kapa2G Robust were the only enzymes that showed efficient amplification of the AT-rich locus in the presence of TMAC additive. Interestingly, the two Kapa enzymes did not produce detectable amplification of this locus in the absence of TMAC, which demonstrates the effect of TMAC on an AT-rich template. PCR amplification of the two test loci were performed at least twice for each enzyme tested and in each case the same results were obtained. Enzymes and PCR conditions that showed some level of amplification of the AT-rich locus were used to amplify genomic libraries for Illumina sequencing. We analyzed the sequence data to assess suitability of each polymerase and conditions for amplification of AT-rich libraries.

### PCR amplification of Illumina genomic libraries

Although Phusion polymerase is the standard enzyme for Illumina sequencing library amplification, it produces libraries with poor representation of loci with extreme base composition particularly the AT-rich regions [4]. In our screening above, many enzymes failed to show efficient amplification of the test locus and were clearly intolerant to AT-rich templates. We evaluated the efficiency of genomic library amplification using polymerases that showed some tolerance to the AT-rich template. We tested Phusion, AccuPrime Taq HiFi, Platinum pfx, Kapa2G Robust and Kapa HiFi. We used these enzymes to amplify PE adapter-ligated genomic libraries made from malaria parasite clinical isolate and the reference strain *P. falciparum *3D7. In the presence of TMAC, AccuPrime Taq HiFi and Phusion produced undetectable amplifications that were not processed further. However, alternative amplification conditions produced high amplification yields (see additional file 1, Figure S1), that were sequenced and analyzed. All sequence data sets generated were normalized to average coverage before analysis. Two data sets were generated in parallel: One from a lab-cultured parasite (*P. falciparum *strain 3D7) genomic DNA; and the other one from *P. falciparum *clinical isolate containing ~53% host DNA contamination. Sequence data from the clinical isolate were filtered to remove host contamination before analysis resulting in lower coverage depths compared to those from 3D7. To analyze and compare the data generated under optimized conditions against standard procedures, we developed analysis metrics that assess four aspects of data quality: Evenness of coverage depth metrics was used to analyze the distribution of coverage depth for each library across the entire genome and plot results of multiple data sets in a single graph for comparative analysis; GC content analysis was used to compute the average GC content for each library and assess base-composition biases; tolerance metrics was used to assess representation of extreme base-composition loci, focusing on selected regions; and fidelity metrics was used to assess enzyme-dependent amplification errors.

**Figure 2:**
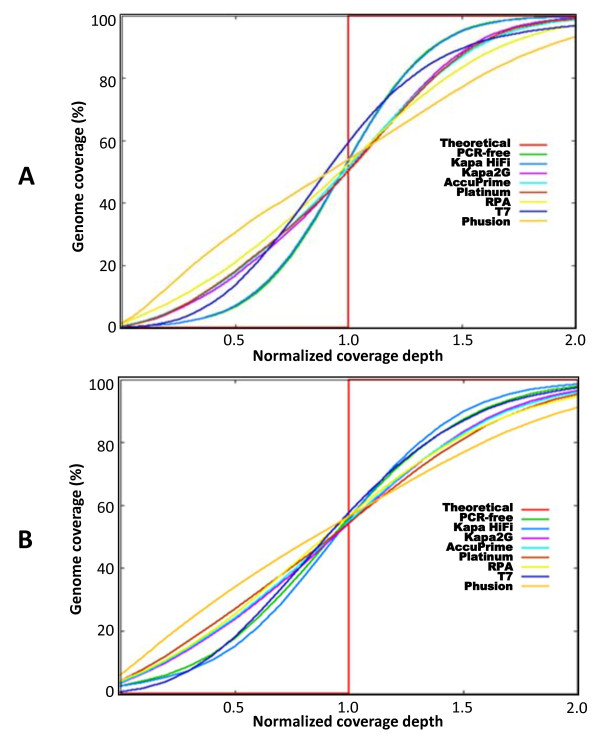
**A plot of genome coverage against normalized average depth**. Duplicate data sets were normalized and pooled. Variance in coverage above and below the normalized average depth (red vertical line) across the genome is shown. Deviation of sample curves from the average depth indicates level of evenness in coverage depth distribution across the genome. The closer the sample curve is to the vertical line, the more even the coverage. The theoretical curve represents average normalized depth at 100% genome coverage. A) Coverage by libraries made from *P. falciparum *3D7 (1 normalized depth represents 21×). B) Coverage by libraries made from clinical isolate, PK0076 (1 normalized depth represents 11×). Kapa HiFi, Kapa2G and Platinum pfx enzymes were used in the presence of TMAC.

**Figure 3:**
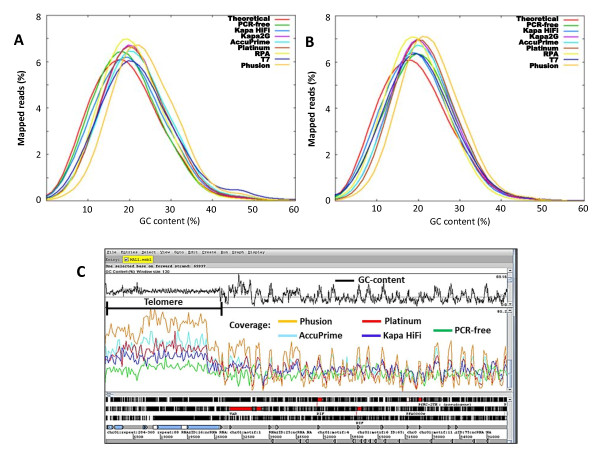
**GC profile analysis of sequenced data**. The GC content distribution for different library preparation methods are shown alongside theoretical data for comparison. A) GC content analysis on libraries prepared from *P. falciparum *3D7 with mapped reads normalized to 21× genome coverage. B) GC content of libraries prepared from a clinical isolate (PK0076) with mapped reads normalized to 11× genome coverage. Libraries with GC content above 19.4% (the GC content of the *P. falciparum *3D7 reference genome) indicate amplification bias towards templates with neutral GC composition. C) Artemis [9,10] screen view of coverage (mapped reads normalized to 21× genome coverage) for a PCR-free library and four other libraries under test on *P. falciparum *3D7 chromosome 1 (zoomed in to show coverage on the GC rich telomere). Kapa HiFi, Kapa2G and Platinum pfx enzymes were used in the presence of TMAC. See additional file 1, Figure S2 A & B for coverage on the entire chromosome 1 and AT-rich locus.

Using these metrics, we show that libraries prepared under standard conditions (using Phusion polymerase) produced less even coverage compared to the optimized conditions in both 3D7 and the clinical isolate. As shown in Figure 2A &2B, up to 30% of the genome was covered at depths of ≤10× for 3D7 and ≤5× for Clinical isolate in Phusion-amplified libraries. In contrast libraries amplified under optimized conditions had most of the genome covered at high depths and only <10% of the genome covered at depths ≤10× and ≤5× for 3D7 and clinical isolate respectively. Libraries prepared under standard conditions also showed strong bias towards the GC-rich templates as shown by a shift in average GC content from ~19.4% (un-amplified reference genome) towards GC neutral base composition (Figure 3A &3B). Table 1 shows the average GC content for each amplification condition tested. To illustrate this further, we used artemis [9,10] to view mapped reads on chromosome 1 and assess coverage depth distribution with respect to base composition as depicted by a GC content plot (see Figure 3C and additional file 1, Figure S2 B). We show that under standard conditions, high GC content regions across chromosome 1 including telomeres were over-amplified whereas regions of high AT content were under-amplified. On the other hand Kapa HiFi amplification was close to that of a PCR-free library in coverage of either extremes of base composition.

**Table 1:** Average GC content

Sample	Library	PCR-free	Kapa HiFi	Kapa2G	AccuPrime	Platinum	RPA	T7	Phusion
**3D7**	Av. %GC content	19.50	20.35	21.44	21.93	21.47	20.75	22.63	23.80
**PK0076**	Av. %GC content	21.92	19.79	21.07	21.37	21.91	19.66	20.95	22.87

Calculated values of average GC content corresponding to each library preparation. Deviation from the 19.4% (value of the unamplified genome) indicates the effect of amplification bias. See Figure 3 A & B for a global graphical presentation of the GC content distribution. 3D7, *P. falciparum *strain 3D7; PK0076, *P. falciparum *clinical isolate.

**Figure 4:**
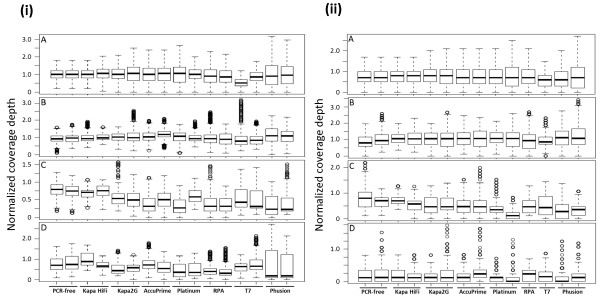
**Box plots showing coverage analysis of *P. falciparum *chromosome 11**. (i) *P. falciparum *3D7; mapped reads normalized to 21× genome coverage (1 normalized depth represents 21×). (ii) Clinical isolate PK0076; mapped reads normalized to 11× genome coverage (1 normalized depth represents 11×). Subplots B, C and D in both i & ii show coverage of sub-regions of the *P. falciparum *3D7 chromosome 11. A) Coverage depth variability plotted for each library on the entire chromosome. B) Distribution of base coverage depth for each library over gene *Pf11_0074 *and its neighboring introns. C) Distribution of base coverage depth at positions 259985-260864 (extreme AT-region). D) Distribution of base coverage depth at positions 29092-30361 (VAR gene and introns). Top and bottom sides of a box plot represent 75^th ^and 25^th ^percentile of base coverage-depth distribution respectively. The middle line represents 50^th ^percentile. A narrow box indicates less variation in coverage depth across that locus and vice versa. Kapa HiFi, Kapa2G and Platinum pfx enzymes were used in the presence of TMAC. All *P. falciparum *3D7and most clinical isolate libraries were prepared in duplicate and each replicate data plotted independently as shown.

We also assessed coverage across chromosome 11 and then focused on specific regions of balanced GC content, high AT content and variable base compositions. A box plot showing variation in depth coverage for both *P. falciparum *strain 3D7 and the clinical isolate is shown on Figure 4i & ii respectively. In these analyses, we observed relatively even coverage depth across the base-balanced region (panel B of Figure 4i & ii) by all the enzymes and conditions tested (this is indicated by the narrow gap between the 25^th ^and 75^th ^percentile of base coverage distribution). However, large variations in coverage depths were observed across the AT-rich and the variable base composition loci (Figure 4i & ii panels C and D respectively). Although most enzymes showed more consistency in coverage depth than Phusion, Kapa HiFi produced the best coverage at the AT-rich regions with evenness almost matching that of PCR-free library. At this AT-rich locus in 3D7 (Figure 4i-C), 75th and 25^th ^percentiles of base coverage depths by both Kapa HiFi libraries produced ~18× and 13× coverage respectively. On the other hand 75th and 25^th ^percentile of base coverage depth by both Phusion libraries produced ~13× and 3× coverage respectively. The wider gap in coverage depth distribution by Phusion-amplified library indicates uneven and poor coverage of AT-rich regions. For all conditions, coverage of positions 29092-30361 (VAR gene and introns) was generally very low and inconsistent in the clinical isolate (Figure 4(ii) D) compared to 3D7 (Figure 4(i) D), perhaps due to the antigenic variation phenomenon associated with these genes. Overall, we show that our optimized conditions improve the quality of coverage in both clinical and non-clinical samples.

### Isothermal exponential amplification of Illumina genomic libraries using RPA technology

High extension temperatures in conventional PCR procedures have been associated with bias and other negative effects on high AT content libraries. We therefore assessed the suitability of an isothermal exponential amplification method provided by the recombinase polymerase amplification (RPA) technology [11]. RPA uses recombinase enzymes which bind oligonucleotide primers (single-stranded DNA) to form nucleoprotein filaments that stimulate searching for homologous sequences in a duplex-DNA template. The primer-recombinase complex is able to attach to the homologous DNA duplex and facilitate strand exchange, eliminating the need to denature the template as in PCR. Disassembly of the recombinase allows strand-displacing DNA polymerase to begin primer extension from the 3' end. Two opposing oligonucleotide primers initiate an isothermal exponential amplification through automatic cyclic repetition of this process. We used RPA technology (TwistDx) with Illumina PE primers to amplify adapter-ligated libraries before sequencing. We obtained a relatively high yield of RPA-amplified library compared to T7 libraries (see additional file 1, Figure S1). Analysis of sequence data obtained from RPA-amplified libraries showed reduced bias towards GC rich templates compared to standard Illumina libraries for both *P. falciparum *3D7 and the clinical isolate (Figure 3A &3B). Coverage of extreme AT base-composition loci was however inferior to Kapa enzymes (Figure 4i & ii panel C). Furthermore, RPA amplified libraries showed relatively high levels of chimeric and duplicate reads (see additional file 1, Table S2).

### Linear amplification of Illumina genomic libraries using T7 RNA polymerase

Exponential amplification of sequencing libraries is thought to be the source of biases and artifacts. To avoid this, we explored a recently described linear amplification for deep sequencing (LADS) protocol [12]. LADS uses T7 RNA polymerase to transcribe DNA fragments bearing a T7-promoter sequence at the 5' end. This is an *in-vitro *transcription system whereby the T7 RNA polymerase transcribes a DNA fragment multiple times producing several RNA transcripts under isothermal conditions and resulting in a linearly amplified template. The resulting RNA material is then converted to cDNA using a reverse transcriptase. We sequenced the T7 polymerase-amplified libraries and analysed the quality of the data generated using our analysis metrics. The overall yield of the library generated using the LADS protocol was very low compared to those amplified exponentially (see additional file 1, Figure S1). However, coverage of extreme base-composition loci was better than standard Illumina libraries. Libraries produced under optimized PCR conditions were generally superior to the LADS protocol (T7) in coverage of AT-rich loci for both *P. falciparum *3D7 and the clinical isolate (Figure 4 I & ii panel C). Surprisingly, an over-representation of fragments with high GC content was observed in T7-amplified library when viewing sequence reads aligned to the reference genome using artemis [10] (data not shown). T7 polymerase-amplified libraries were also associated with the highest level of duplicate and chimeric reads (see additional file 1, Table S2).

### Effect of TMAC on Illumina genomic library amplification

TMAC has been shown to improve thermostability of AT-base pairs [7]. However, it inhibits the activity of most polymerases. In our analysis, Phusion and AccuPrime Taq HiFi were inhibited by TMAC and produced no library amplification (see additional file 1, Figure S1). On the other hand Platinum pfx and the Kapa enzymes were tolerant to the concentration of TMAC used in this assay (Figure 1). We analyzed and compared libraries prepared with Kapa enzymes in the presence and absence of TMAC. Our analysis indicates that the overall evenness in coverage across the genome was significantly improved in the presence of TMAC (see additional file 1, Figure S3). We observed less bias towards GC rich sequences and an overall improved coverage on AT-rich regions. All amplification data shown for Kapa HiFi, Kapa2G and Platinum pfx in Figures 2, 3 and 4 were performed in the presence of TMAC. The positive effect of TMAC was observed in both *P. falciparum *3D7 and the clinical isolate (containing ~53% host contamination).

### Fidelity and coverage ranking

We analyzed the accuracy of each enzyme in terms of base incorporation by counting mismatches and indels at regions of low complexity (TATA repeats). At these regions of low complexity, RPA generated the least number of errors (scoring best) whereas T7 polymerase had the highest (Table 2). Kapa HiFi showed a relatively low mismatch score which was largely due to high number of insertions observed at TATA repeat regions where most of the enzymes tested showed little or no coverage. Enzymes were also scored on evenness of coverage depth by counting the number of regions with zero and less than 5× base coverage. Based on these criteria, Phusion had the highest number of regions with zero and <5× coverage, thereby scoring poorest. Kapa HiFi had the best coverage score after PCR-free. Finally, we calculated the overall fidelity and evenness of genome coverage and ranked each amplification condition based on average overall score. As shown in Table 2, libraries amplified by Kapa HiFi came second to PCR-free, whereas Phusion polymerase had the lowest overall score (see additional file 1, Table S3 for raw data used in ranking).

**Table 2:** Ranking of *P. falciparum *3D7-amplified and PCR-free libraries.

Amplification	Coverage score	MM score	Rank ALL
PCR -Free	0.99	0.95	0.97
Kapa HiFi	0.8	0.58	0.69
Platinum pfx	0.67	0.59	0.63
AccuPrime Taq HiFi	0.59	0.56	0.58
RPA	0.52	0.6	0.56
Kapa2G Robust	0.59	0.57	0.58
Phusion	0.22	0.5	0.36
T7	0.74	0.43	0.58

Ranking was based on genome coverage and accuracy. Coverage score: PCR-free and Kapa HiFi had the lowest percentage of genome covered at 0 or <5× thereby scoring the highest. Mismatch (MM) score: Loci of low complexity were used to generate mismatch ranking (see Material and Methods under Fidelity section). The number of True positive mismatches, False positive mismatches, Deletion and Insertions obtained for each library were used to generate a score before combining all scores to generate an overall mismatch rank. Using these criteria, RPA produced the highest score and T7 scored the least. See additional file 1, Table S3 for raw ranking data.

## Discussion

Library amplification is an important step in preparing samples for sequencing with the current NGS platforms, especially when the starting material is severely restricted in quantity. PCR amplification, however, has been shown to be the major source of artifacts and base-composition bias in library fragments. Although alternative library-preparation approaches that omit the PCR amplification show a significant reduction in base-composition bias, this strategy requires large quantities of starting material. Therefore, PCR-free library preparation is unsuitable where amounts of DNA starting material are limited, as is the case with most clinical isolates.

We demonstrate that problems associated with PCR can be ameliorated through optimization, thereby allowing amplification to be used in generating sequencing libraries even from extremely AT-biased genomes. Through careful selection of polymerases and the use of a PCR-additive that normalizes melting temperatures of the different library fragments, we have optimized Illumina library amplification. These alternative PCR conditions generate library fragments with increased coverage of extreme AT-rich regions.

Standard Illumina amplification procedures that use Phusion polymerase generate libraries with biased base-composition and are depleted of AT-rich loci. The sequence data generated under these standard conditions are of reduced complexity and maybe unsuitable for resequencing to identify relatively rare genetic variants of important biological function. We set out to solve this problem by focusing on the library amplification step, which is the primary source of biases in almost all NGS platforms. We started by screening commercially available polymerases for tolerance to an extremely AT-rich template. This initial screening procedure identified a preferred polymerase and amplification conditions that showed improved tolerance to AT-rich templates. We demonstrated that most commercial polymerases are inhibited by TMAC. However, this chemical can have a tremendous positive effect in amplifying AT-rich templates for the TMAC-tolerant enzymes. With TMAC additive we have developed Illumina library-amplification protocol suitable for extreme AT-rich genomic DNA. Quality analyses of sequence data from both *P. falciparum *3D7 and a clinical isolate generated by our optimized conditions showed less bias towards base-balanced regions and produced an average GC content that matches that of the unamplified genome.

To date, no single polymerase or amplification method has been shown to efficiently amplify both extremes of base composition with equal and optimum efficiency. The two isothermal amplification methods (RPA and T7) tested here showed some level of improved coverage relative to standard amplifications, but they were both associated with high levels of chimeric and duplicate reads. Under the optimized conditions we show that the Kapa series of polymerases in the presence of TMAC produced the best coverage that was relatively consistent across regions of extreme base composition. Worthy of note is the low template-dependent fidelity score for Kapa HiFi (Table 2), where we detected a number of false-positive insertion calls mainly in regions of TATA repeats. Other enzymes had zero or extremely low coverage in these regions, limiting our ability to perform accurate comparative analysis on enzyme's fidelity at very low complexity regions.

## Conclusion

Despite the bias problem associated with it, PCR still remains an essential step in many NGS library preparation procedures, especially when the starting DNA material is limited. We describe robust alternative PCR conditions optimized for extremely AT-biased genomes. We show that the optimized conditions significantly reduce bias and also retain the complexity of either extremes of base composition. This observation was consistent in both the lab-cultured strain (3D7) and the clinical isolate. The optimized procedures described here will greatly benefit the malaria genomic research and control initiatives that have faced serious challenges with the parasite's extreme AT-rich genome.

## Methods

### Genomic DNA

*P. falciparum *3D7 genomic DNA was a gift from Prof Chris Newbold (University of Oxford). Other *P. falciparum *genomic DNA (PK0076) was a clinical isolate containing ~53% human DNA contamination. The clinical isolate was obtained from the Malaria Genetics Group's Sequencing Sample Repository at the Wellcome Trust Sanger Institute.

### Screening for an AT-rich tolerant enzyme

Various commercial enzymes were used to perform a conventional PCR (50 µl total volume) targeted at a 1217 bp locus (Pf3D7_01:55900-57116) with a high AT-content (84%) and a 540 bp locus (Pf3D7_11:1294982-1295521) with a relatively balanced base composition (positive control). Ten ng of *P. falciparum *3D7 genomic DNA was used as template for each PCR. Pf_1 and Pf_11_ama1 oligo pairs (see additional file 1, Table S1) were used at a final concentration of 0.3 µM to amplify the high AT-rich and positive control fragments respectively. For each enzyme, two sets of PCRs were performed, one using reagents and conditions provided by the manufacturer (standard conditions) and the other deviating only by inclusion of 60 mM TMAC in the reaction mixture (optimized conditions). Each set of amplification condition was repeated at least twice to confirm the finding. See additional file 1, Table S4 for enzymes used and their catalogue numbers.

### Illumina sequencing libraries

Genomic DNA (0.05 - 1 μg in 75 µl TE buffer) from *P. falciparum *3D7 or the clinical isolate was sheared for 3 min using a Covaris S2 (Covaris Inc., Woburn, MA).To obtain a fragment-size distribution of ~100 bp to ~300 bp, the settings were: 20% duty cycle, intensity 5, and 200 cycles per burst. Illumina paired-end sequencing libraries were constructed using the NEBNext DNA sample preparation kit (NEB), following standard Illumina sample-preparation protocol with the following slight modifications. End-repair reactions (100 μl) contained 75 µl of sheared DNA sample, 1× T4 DNA-ligase buffer, 1 mM ATP, 0.4 mM dNTPs, 15 units of T4 DNA polymerase, 50 units of Klenow DNA polymerase, 50 units of T4 polynucleotide kinase, and were incubated at 20°C for 45 min. Resulting blunt phosphorylated DNA fragments (end-repaired) were cleaned using the QIAquick PCR purification Kit following the manufacturer's instructions and the DNA eluted in 32 μl of buffer EB. A single A base was added (A-tailing) to the 3' end of the end-repaired DNA fragments in a 50 μl reaction containing 1× Klenow buffer, 0.2 mM dATP, 150 units of Klenow exo- and incubated for 45 min at 37°C. A-tailed DNA fragments were cleaned using QIAquick MiniElute PCR purification Kits following the manufacturer's instructions and DNA eluted in 10 µl EB buffer. Pre-annealed paired-end (PE) adapter oligonucleotides (Illumina) were ligated to the A-tailed fragments in a 50 µl reaction containing 10 µl of DNA sample, 1× Quick T4 DNA-ligase buffer, 10 µl of PE-adapter mixture, 5 µl of Quick T4 DNA ligase (NEB) and incubated at 20°C for 30 min. The ligation reaction was cleaned twice with 1× Agencourt Ampure XP beads (Beckman Coulter). Aliquots were analyzed by running on an Agilent 2100 Bioanalyzer (Agilent Technologies) to determine the size distribution and to check for adapter contamination. Most amplified or non-amplified libraries were prepared twice (starting from same non-sheared genomic DNA) and each technical replicate sequenced independently before data analysis. All replicate data sets were pooled after normalizing and before analysis with the exception of coverage analysis on specific genome loci (presented as box plots, Figure 4i &4ii) where each replicate was analyzed and shown independently.

### Illumina PCR library amplification

All PCR amplifications were performed with an MJ Research Thermo Cycler PTC-225. Illumina PE 1.0 and 2.0 primers or PE 1.0- and 2.0-derived indexing primers were used to amplify 10 ng adapter-ligated library fragments by PCR. From a single pool of adapter-ligated library, aliquots were amplified using standard Illumina PCR reagents or using various alternative polymerases under optimized PCR conditions. Standard Illumina PCR (50 μl) with Phusion polymerase (Thermo) contained 1× Phusion DNA polymerase master mix and 0.4 μM of each primer pair and was amplified with thermocycling conditions of 1 min at 98°C for the initial denaturation, followed by 12 cycles of [10 s at 98°C, 30 s at 65°C, 30 s at 72°C] and a final extension for 5 min at 72°C. PCR (50 μl) with Kapa HiFi (KAPA Biosystems, South Africa) contained 1× Kapa HiFi buffer (containing TMAC) or 1× Kapa buffer B, 0.3 mM of each dNTP, 0.4 μM of each primer pair, 1 unit of Kapa HiFi and was amplified with thermocycling conditions of 1 min at 98°C for the initial denaturation followed by 12 cycles of [10 s at 98°C, 1 min at 65°C] and a final extension for 5 min at 65°C. PCR (50 μl) with Kapa2G Robust (KAPA Biosystems, South Africa) contained 1× Kapa2G buffer B, 0.3 mM dNTPs mix, 0.4 μM of each primer pair, 0.4 units of Kapa2G Robust and was amplified in the presence or absence of 60 mM TMAC with the same thermocycling conditions as Phusion above. PCR (50 μl) with Platinum pfx (Invitrogen) contained 1× Platinum pfx buffer, 0.3 mM dNTPs mix, 0.4 μM of each primer pair, 1 mM MgCl_2_, 0.8 units of Platinum pfx and was amplified in the presence or absence of 60 mM TMAC with thermocycling conditions of 2 min at 94°C for the initial denaturation followed by 12 cycles of [15 s at 94°C, 30 s at 60°C, 30 s at 65°C] and a final extension for 5 min at 65°C. PCR (50 μl) with AccuPrime Taq HiFi (Invitrogen) contained 1× AccuPrime buffer II, 0.4 μM of each primer pair, 1 unit of AccuPrime Taq HiFi and was amplified in the presence or absence of 60 mM TMAC with the thermocycling conditions used for Platinum pfx. PCR products were purified with 1× Agencourt Ampure XP beads (Beckman Coulter) and eluted in EB buffer (Qiagen). PCR products were analysed by running on an Agilent 2100 Bioanalyzer (Agilent Technologies) to assess amplification. Only successful amplifications were processed for sequencing.

### Illumina isothermal library amplification using RPA technology

We used recombinase polymerase amplification (RPA) technology as provided in the TwistAmp™ DNA-amplification kit [11] to amplify 10 ng adapter-ligated library fragments. Illumina PE 1.0 and 2.0 primers or PE 1.0- and 2.0-derived indexing primers (each in 10 μM stock) were used for amplification following kit manufacturer's instructions. Amplifications were performed twice to generate technical replicates. For each sample, a rehydration solution was prepared by mixing 2.4 μl of each primer pair, 29.5 μl of rehydration buffer and 13.2 μl of template library solution. After vortexing and brief centrifugation, the 47.5 μl of rehydration solution was transferred to a freeze-dried RPA reaction complex pellet and mixed by pipetting up and down until complete resuspension was achieved. To initiate the reaction, 2.5 μl of 280 mM magnesium acetate was added and mixed well by vortexing followed by brief centrifugation. The reaction was allowed to continue in an incubator block at an isothermal temperature of 37°C for 40 min. Amplification products were purified with 1× Agencourt Ampure XP beads (Beckman Coulter) and eluted in EB buffer (Qiagen).

### Illumina linear library amplification using T7 RNA polymerase

Linear amplification for deep sequencing (LADS) [12] was used to amplify end-repaired and A-tailed genomic DNA library fragments. Starting with 200 ng of genomic DNA, ~30 ng of A-tailed library fragments were ligated to Adapter-A and Adapter-B, previously prepared by annealing adapter-A and adapter-B forward and reverse oligos (see additional file 1, Table S1) respectively. Adapter-ligated libraries were cleaned twice with 1× Agencourt Ampure XP to remove non-ligated adapters and adapter dimers. Alternatively, adapter-ligated library was electrophoresed in a 2% agarose gel alongside a DNA ladder and fragment bands of 250 to 600 bp were cut and purified at room temperature using the Qiaquick gel extraction kit (Qiagen). The purified adapter-ligated library (20 ng) was used to perform *in vitro *transcription at 37°C overnight with reagents from the MEGAscript T7 kit (Ambion). The protocol described in [12] was followed to generate T7-amplified libraries ready for Illumina cluster generation and sequencing. The procedure was repeated twice, each time starting from same genomic DNA, to generate technical replicates

### PCR-free libraries

Genomic DNA (1-2 μg) was sheared, end-repaired and A-tailed as described under "Illumina sequencing libraries". Pre-annealed paired-end Illumina No-PCR adapters were ligated using the same ligation procedure described under "Illumina sequencing libraries". Resulting adapter-ligated fragments were loaded alongside a low molecular weight ladder (NEB) on a TBE 2% agarose gel. Fragment bands of 350 to 500 bp were cut and purified using a Qiaquick gel extraction kit. Gel purification was performed at room temperate and DNA eluted in EB buffer.

### Library quantification by qPCR

Pre-PCR libraries were quantified by qPCR using a previously quantified and sequenced library in dilutions containing 100 ng, 10 ng and 1 ng as standard. The qPCR reaction (25 μl) contained 12.5 μl of 2× Sybr Green PCR Master Mix (Applied Biosystems), 0.75 μl of 10 μM Pre-PCR qPCR top and bottom primers (see additional file 1, Table S1), 2.5 μl of library sample (pre-diluted 1000 fold) and 8.5 μl of PCR-quality water. The reaction was set in triplicates and run on a StepOne™ Real-Time PCR System (Applied Biosystems) with a thermocycling protocol of 10 min at 95°C for the initial denaturation followed by 40 cycles of [30 s at 95°C and 1 min at 60°C]. Prior to cluster generation, amplified or no-PCR libraries were quantified by qPCR using the SYBR fast Illumina library quantification kit (KAPA Biosystems). The qPCR was done following instructions provided by the kit manufacturer and run on the StepOne™ Real-Time PCR System (Applied Biosystems). The thermocycling protocol was 5 min at 95°C for the initial denaturation followed by 35 cycles of [30 s at 95°C and 45 s at 60°C].

### Data processing

Replicate data sets were analyzed independently by mapping sequence reads to the *P. falciparum *3D7 reference genome V2.1.5 ftp://ftp.sanger.ac.uk/pub/pathogens/Plasmodium/falciparum/3D7/3D7.version2.1.5/ using BWA [13]. SAMtools [14] was used to generate coverage data from the BWA pileup mapping output. To compare the quality of sequence data from different library preparation methods, we developed four analysis metrics. Evenness of coverage depth metrics - comparing the overall representation and depth across the entire genome; GC content analysis - to detect base composition biases in amplification through comparison with a theoretical GC content derived from shredded reads of the reference genome [6]; Tolerance metrics - to assess representation of extreme base-composition loci, focusing on selected genomic regions; and Fidelity metrics - assessing enzyme-dependent errors.

### *Genome coverage*

Mapped reads for each replicate data sets were normalized by average genome coverage to 11× and 21× for clinical and non-clinical samples respectively, and then pooled or analyzed independently depending on analysis metrics used. For the clinical samples, reads originating from host contamination were filtered out (by aligning all reads to *P. falciparum *3D7 reference genome) prior to analysis, hence the relatively low average genome coverage depth for these samples. Evaluation of coverage depth and evenness was based on cumulative distributions over a normalized overall average depth. A measurement of low-coverage index lci (*d*) is defined as the integration of the cumulative distribution C(x) from 0 to *d *giving an overall assessment of the coverage at the low end of distribution:

$$lci\left(d\right)=\int_{0}^{d}C\left(x\right)dx$$

The value lci (0.5) that gives a measurement of the coverage below half of the average depth in the distribution was used to compare evenness of coverage for each data set.

We counted the number of bases in the genome with zero coverage (Cov = 0) and less than 5× coverage (Cov<5×). We used SAMtools to generate coverage plots and bash/awk scripts for coverage counting. Tolerance to regions of extreme base composition was analyzed after normalizing data sets by overall average coverage depth. Coverage distribution on selected regions (on chromosome 11) are presented as a boxplot generated in R [15]http://www.r-project.org/.

### *Fidelity*

Most polymerases introduce errors during base incorporation in regions of low complexity. To assess these forced errors (or template-dependent errors), we compared variant calls by each amplification method at sites of low complexity (mainly TATA repeats). We also assessed the ability of each amplification condition to find a set of known consensus errors in the reference, and used data obtained to score true and false positives. The known consensus errors in the reference genome were previously identified and published [16]. We used the iCORN program [16] to conservatively map reads to the reference genome. iCORN iteratively maps reads and compares them with perfectly mapping reads. PCR-free variant calls were included as a quality control. A mismatch or indel was called with mpileup (a function in SAMtools) at a quality ≥40 from the vcf file. We differentiate between a true positive mismatch or indel (if the call agrees with iCORN and PCR-free), and a false positive, (if the call disagrees with iCORN and PCR-free) at that position. Using the total number of errors/variants counted per sample, we generated a normalized error score for each enzyme or library-preparation method.

### Rank

We ranked the results by assigning a score of 1 to the best and a score of 0 to the worst. For each amplification condition, we calculated the average score value obtained in both coverage and fidelity metrics. Conditions with the highest average score was ranked highest while that with the least assigned the lowest rank (see Table 2).

### Chimeric and duplicate reads analysis

We used the BWA paired-end alignment algorithm to generate mapped data sets. Picard software http://picard.sourceforge.net/command-line-overview.shtml was used to identify/mark duplicates from the mapped data set. Chimeric reads were identified from the alignment results using the “bwasw” option with single-end reads. Each replicate data set was analyzed independently to determine the number of chimeric or duplicate reads. We calculated the number of chimeric or duplicate reads as a percentage of the total mapped reads for each data set. Mean values are shown (see additional file 1, Table S2).

All datasets used in this study have been deposited in the ENA read archive under accession number [ERP000832].

## Abbreviations

TMAC: Tetramethyammonium chloride; PCR: Polymerase Chain Reaction; NGS: Next-Generation Sequencing; PE: Paired-end; qPCR: quantitative PCR; RPA: Recombinase Polymerase Amplification; LADS: Linear amplification for deep sequencing; SNP: Single Nucleotide Polymorphism.

## Competing interests

The authors declare that they have no competing interests.

## Authors' contributions

Research in the lab was carried out by SOO, MAQ and SC. Bioinformatic analyses were carried out by TDO, YG, GM and MM. The research was coordinated by BM and HPS. DPK, DJT, HPS and MAQ conceived the project. SOO wrote the paper. All authors read and approved the final manuscript.

## Supplementary Material

Additional file 1 **Figure S1. Bioanalyzer quantification and analysis of libraries**. Aliquots of the PE-adapter-ligated library (shown) was amplified using enzymes/conditions indicated. After purification with Agencourt Ampure XP beads, library products were analyzed using Bioanlyzer. Bioanalyzer traces show various yields obtained by different conditions and amplification methods used. T7 and PCR-free produced the lowest yield. Phusion and AccuPrime amplifications were undetectable in the presence of TMAC (Tetramethylammonium chloride). Additional file 1, **Figure S2. Artemis screen view of coverage**. Effect of GC content on coverage for a PCR-free library and four other amplified libraries under test with *P. falciparum *3D7 chromosome 1. A) Coverage over the entire chromosome. B) Coverage over high AT-content locus. Kapa HiFi and Platinum pfx libraries shown were amplified in the presence of TMAC. (See Figure 3C for coverage of the GC rich telomere). Additional file 1, **Figure S3. Box plots showing coverage analysis of *P. falciparum *chromosome 11**. The effect of TMAC on Kapa HiFi and Kapa2G library amplification. A) Coverage plot for each library on the entire chromosome. Subplots B, C, and D shows coverage of sub-regions of the *P. falciparum *3D7 chromosome 11. B) Shows base coverage distribution for each library over gene *Pf11_0074 *and its neighboring introns. C) Coverage at positions 259985-260864 (extreme AT-region). D) Coverage at positions 29092-30361 (VAR gene and introns). Additional file 1, **Table S1**. **Oligonucleotides used in this study**. *Phosphorothioate linkages protect the overhanging thymidine from exonuclease activity. Additional file 1, **Table S2. Chimera and duplicate analysis**. The average number of chimeric or duplicate reads identified from mapping results is shown for each data set. Percentage of mapped data that were found to be chimeric or duplicated in each data set is shown. Mapped reads were normalized to 21× genome coverage. The two isothermal amplifications generated the highest number of chimeric and duplicate reads. Mapped reads were lower for the duplicate analysis than the chimera analysis because of the different alignment methods used. Additional file 1, **Table S3. Ranking analysis**. Raw ranking data for *P. falciparum *3D7 genome coverage and accuracy scores. Mapped reads were normalized to 21× genome coverage. The number of True positive mismatches, False positive mismatches, Deletion and Insertions identified in each library data sets were used to generate a score before combining all scores to generate an overall mismatch rank. Cov, coverage; MM, mismatch; TP, true positive; FP, false positive; DEL, deletion; IN, insertion. Additional file 1, **Table S4. Commercial DNA polymerases used and their catalogue number**. Click here for file
