# Supplementary material for: Optimizing illumina next-generation sequencing library preparation for extremely at-biased genomes
Source: BMC Genomics. 2012 Jan 3;13:1. doi: 10.1186/1471-2164-13-1 (PMC3312816; doi:10.1186/1471-2164-13-1)
Supplement: Additional file 1 — Figure S1. Bioanalyzer quantification and analysis of libraries. Aliquots of the PE-adapter-ligated library (shown) was amplified using enzymes/conditions indicated. After purification with Agencourt Ampure XP beads, library products were analyzed using Bioanlyzer. Bioanalyzer traces show various yields obtained by different conditions and amplification methods used. T7 and PCR-free produced the lowest yield. Phusion and AccuPrime amplifications were undetectable in the presence of TMAC (Tetramethylammonium chloride). Additional file 1, Figure S2. Artemis screen view of coverage. Effect of GC content on coverage for a PCR-free library and four other amplified libraries under test with P. falciparum 3D7 chromosome 1. A) Coverage over the entire chromosome. B) Coverage over high AT-content locus. Kapa HiFi and Platinum pfx libraries shown were amplified in the presence of TMAC. (See Figure 3C for coverage of the GC rich telomere). Additional file 1, Figure S3. Box plots showing coverage analysis of P. falciparum chromosome 11. The effect of TMAC on Kapa HiFi and Kapa2G library amplification. A) Coverage plot for each library on the entire chromosome. Subplots B, C, and D shows coverage of sub-regions of the P. falciparum 3D7 chromosome 11. B) Shows base coverage distribution for each library over gene Pf11_0074 and its neighboring introns. C) Coverage at positions 259985-260864 (extreme AT-region). D) Coverage at positions 29092-30361 (VAR gene and introns). Additional file 1, Table S1. Oligonucleotides used in this study. *Phosphorothioate linkages protect the overhanging thymidine from exonuclease activity. Additional file 1, Table S2. Chimera and duplicate analysis. The average number of chimeric or duplicate reads identified from mapping results is shown for each data set. Percentage of mapped data that were found to be chimeric or duplicated in each data set is shown. Mapped reads were normalized to 21× genome coverage. The two isothermal amplifications generated [file 1471-2164-13-1-S1.PDF]

Additional file 1, Figure S1

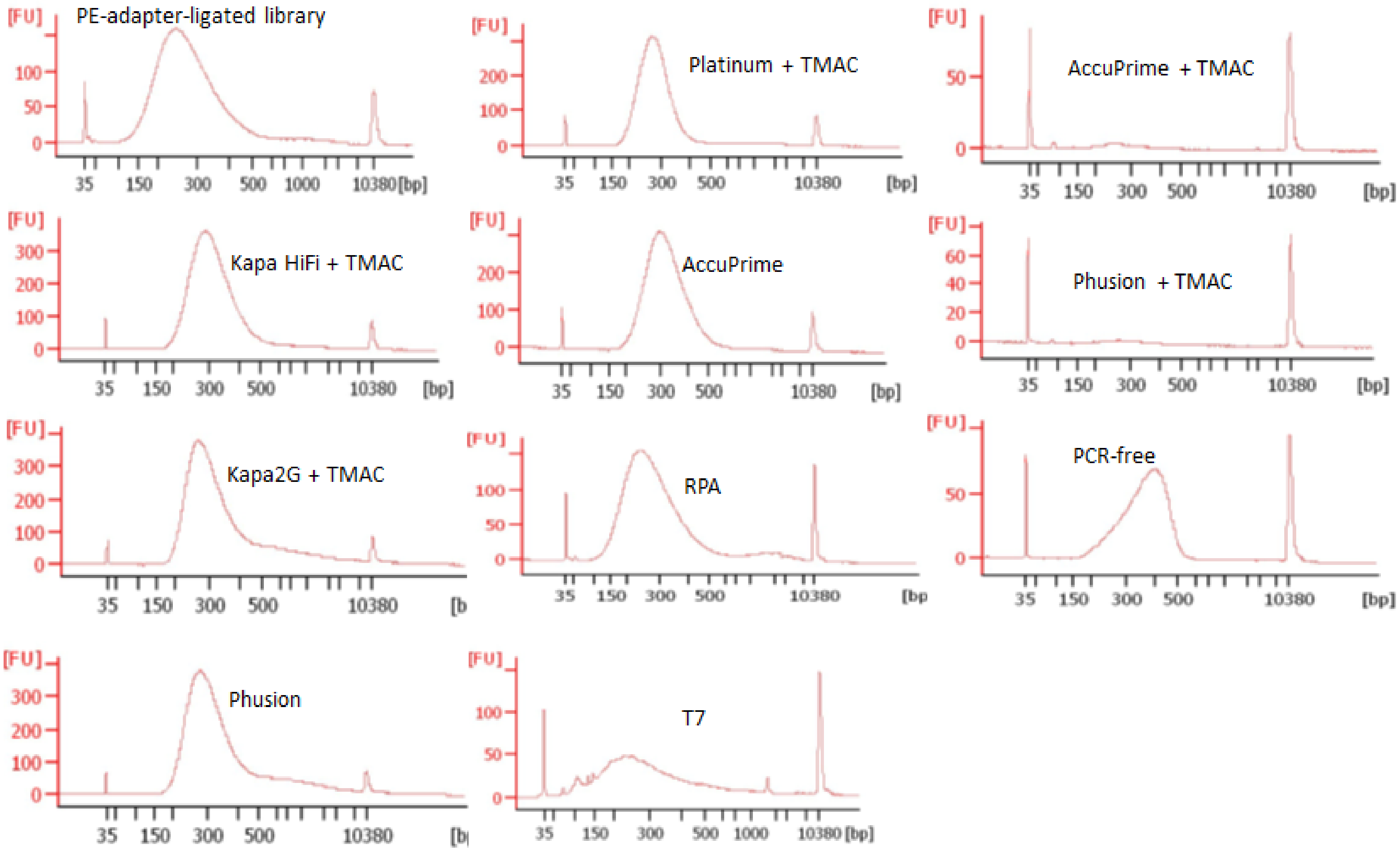

Additional file 1, Figure S2

A

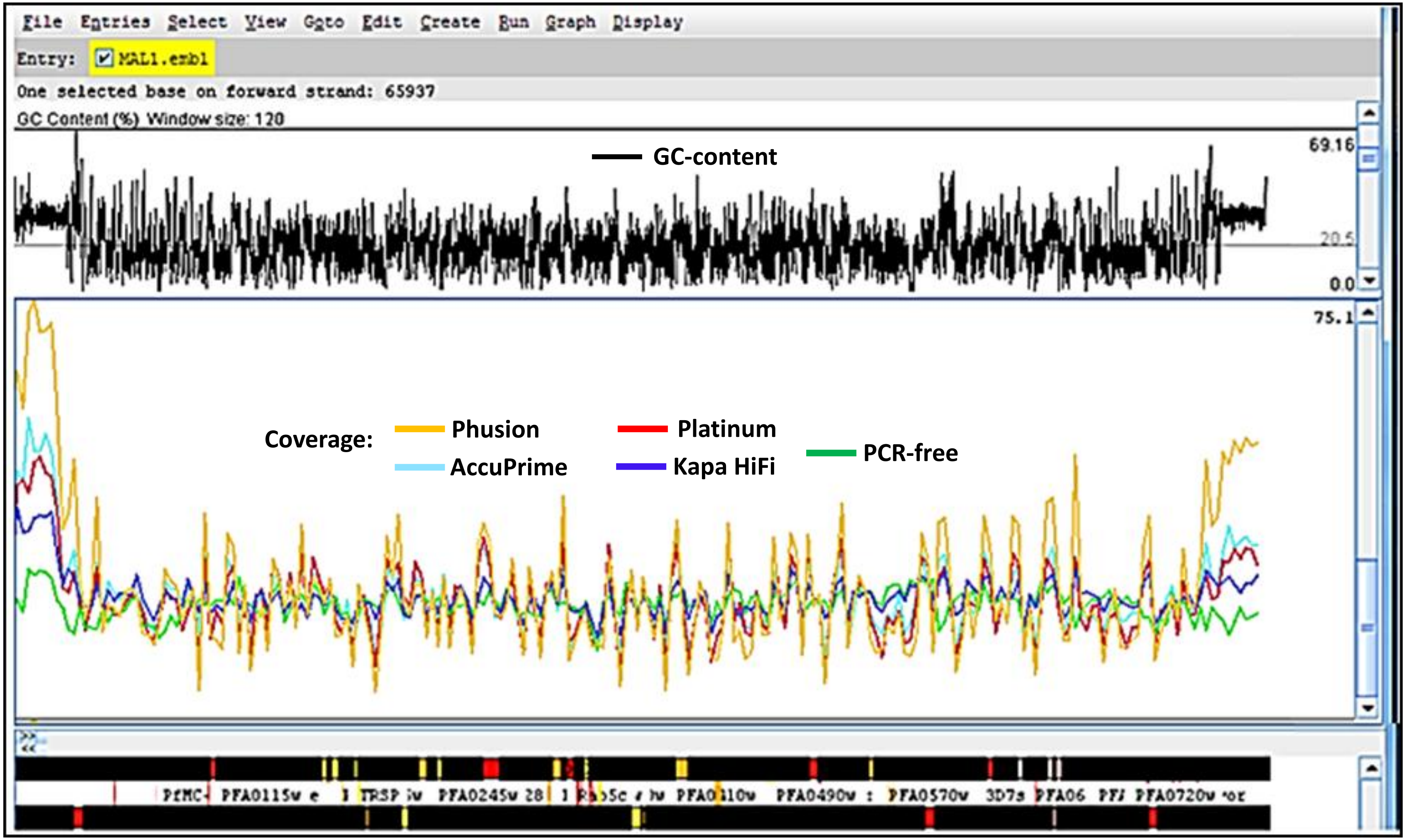

Additional file 1, Figure S2

B

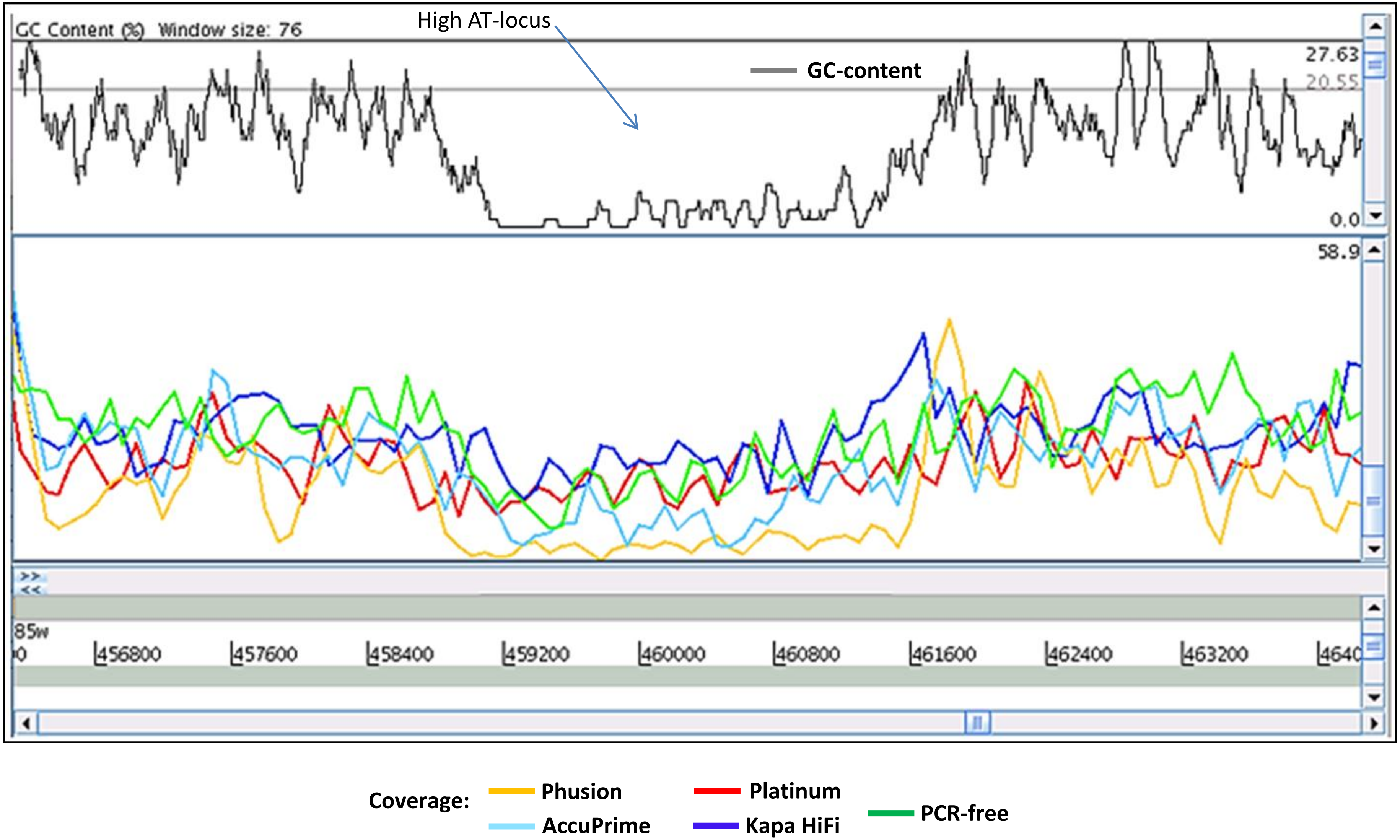

Additional file 1, Figure S3

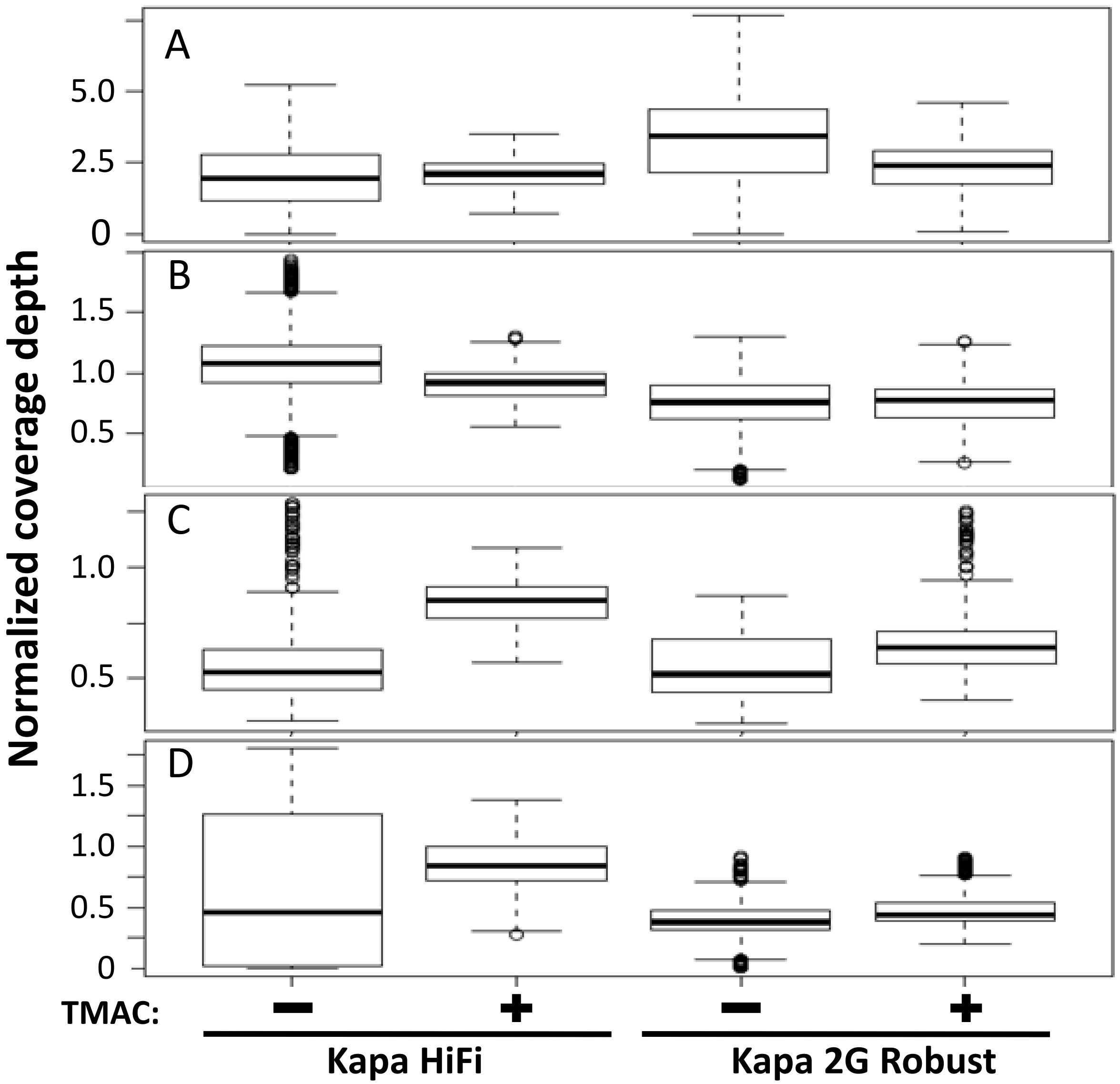

Additional file 1, Table S1

Name	Sequence (5’-3’)
**Pf_1-fwd**	CTCTCTAATTTATCTACGTAG
**Pf_1-rev**	ATGCATGATATTGGAGTGGCAC
**Pf_11_ama1_fwd**	AGTGCTTTTCTTCCCACTGGTGCTT
**Pf_11_ama1_fwd**	AGCGACAGCAGCTGATGATGCAA
**Adapter-A_forward**	AATGATACGGCGACCACCGAGATCTACACTCTTTCCCTACACGACGCTCTTCCGATC*T
**Adapter-A_reverse**	GATCGGAAGAGCGTCGTGTAGGGAAAGAGTGTAGATCTCGGTGGTCGCCGTATCATT
**Adapter-B_forward**	GAATTTAATACGACTCACTATAGGGACAAGCAGAAGACGGCATACGAGATCGGTCTCGGCATTCCTGCTGAACCGCTCTTCCGATC*T
**Adapter-B_reverse**	GATCGGAAGAGCGGTTCAGCAGGAATGCCGAGACCGATCTCGTATGCCGTCTTCTGCTTGTCCCTATAGTGAGTCGTATTAAATTCAATGAT ACGGCGACCACCGA
**Pre-PCR-qPCR-T**	CTTTCCCTACACGACGCTCTTC
**Pre-PCR-qPCR-B**	ATTCCTGCTGAACCGCTCTTC

Additional file 1, Table S2

Enzyme	Chimeras	Mapped reads	%Chimeras	Duplicates	Mapped reads	%Duplicates
PCR-Free	145020	33905223	0.43	463264	33036814	1.4
Kapa HiFi	51160	13959138	0.37	283744	13859858	2.0
Kapa2G Robust	44041	17979489	0.25	331824	17728728	2.0
AccuPrime Taq HiFi	38039	16172005	0.24	234480	15792266	1.5
Platinum pfx	412541	58824642	0.70	1788607	58518211	3.0
RPA	527936	45674837	1.16	6008333	43899011	14.0
T7	273944	11615356	2.32	1670096	9405924	18.0
Phusion	196960	29545281	0.54	843874	29104626	2.7

Additional file 1, Table S3

Enzyme	% genome Cov=0	Score Cov=0	% genome Cov<5X	Score Cov<5X	MM_TP	Score MM_TP	MM_FP	Score MM_FP	DEL_TP	Score DEL_TP	IN_FP	Score IN_FP
**PCR-Free**	0.22	0.99	1.08	0.99	1234.50	0.95	0.00	1.00	390.00	0.84	0.00	1.00
**Kapa HiFi**	0.97	0.70	2.18	0.91	1138.50	0.82	28.50	0.48	378.50	0.81	4207.50	0.22
**Platinum pfx**	1.00	0.69	5.91	0.65	1016.00	0.65	26.00	0.53	230.00	0.40	1215.50	0.78
**AccuPrime Taq HiFi**	1.41	0.52	5.70	0.66	1012.50	0.65	37.00	0.33	209.00	0.34	348.00	0.94
**RPA**	1.38	0.53	7.95	0.51	811.00	0.38	8.00	0.86	166.00	0.22	179.50	0.97
**Kapa 2G Robust**	1.49	0.49	5.24	0.70	1035.00	0.68	36.00	0.35	239.50	0.42	852.00	0.84
**Phusion**	1.92	0.32	13.38	0.13	759.50	0.31	23.50	0.57	156.50	0.19	313.50	0.95
**T7**	0.48	0.89	6.87	0.58	829.50	0.40	47.00	0.15	149.50	0.17	55.00	0.99

Additional file 1, Table S4

Enzyme	Supplier	Catalogue Number
Pfu Ultra	Agilent	600390-51
Accuprime pfx SuperMix	Invitrogen	12344-040
AccuPrime Taq HiFi	invitrogen	12346-086
Platinum pfx	Invitrogen	11708-021
Platinum Taq HiFi	Invitrogen	11304-029
Kapa HiFi	Kapa Biosystems	KK2601
Kapa2G Robust	Kapa Biosystems	KK5701
Pwo master	Roche	03 789 403 001
Ex Taq	Takara	RR001A
Phusion	Thermo	F-540L
RPA	TwistDx	TWIE1
